# Factors that Affect the Content of Cadmium, Nickel, Copper and Zinc in Tissues of the Knee Joint

**DOI:** 10.1007/s12011-016-0927-5

**Published:** 2017-01-09

**Authors:** Wojciech Roczniak, Barbara Brodziak-Dopierała, Elżbieta Cipora, Agata Jakóbik-Kolon, Joanna Kluczka, Magdalena Babuśka-Roczniak

**Affiliations:** 1 Medical Institute, The Jan Grodek Higher Vocational State School, 21 Mickiewicza Str. 38-500, Sanok, Poland; 2 School of Pharmacy with the Division of Laboratory Medicine, Department of Toxicology, Medical University of Silesia, 4 Jagiellonska Str. 41-200, Sosnowiec, Poland; 3 Faculty of Chemistry, Department of Inorganic, Analytical Chemistry and Electrochemistry, Silesian University of Technology, 6 B. Krzywoustego Str. 44-100, Gliwice, Poland

**Keywords:** Cadmium, Nickel, Copper, Zinc, Knee joint, Factors

## Abstract

Osteoarthritis causes the degradation of the articular cartilage and periarticular bones. Trace elements influence the growth, development and condition of the bone tissue. Changes to the mineral composition of the bone tissue can cause degenerative changes and fractures. The aim of the research was to determine the content of cadmium (Cd), nickel (Ni), copper (Cu) and zinc (Zn) in the tibia, the femur and the meniscus in men and women who underwent a knee replacement surgery. Samples were collected from 50 patients, including 36 women and 14 men. The determination of trace elements content were performed by ICP-AES method, using Varian 710-ES. Average concentration in the tissues of the knee joint teeth amounted for cadmium 0.015, nickel 0.60, copper 0.89 and zinc 80.81 mg/kg wet weight. There were statistically significant differences in the content of cadmium, copper and zinc in different parts of the knee joint. There were no statistically significant differences in the content of cadmium, nickel, copper and zinc in women and men in the examined parts of the knee joint. Among the elements tested, copper and nickel showed a high content in the connective tissue (the meniscus) compared to the bone tissue (the tibia and the femur).

## Introduction

Osteoarthritis is the most common disorder of the locomotor system and affects mainly the elderly. In 80% of people over the age of 60, radiographs show degenerative changes, and 20% of them suffer from pain and limited mobility. Osteoarthritis may also occur in younger people, before the age of 50, and is considered a serious health problem [1]. It is estimated that approximately 40% of degenerative changes of the knee joint is related to body ageing, whereas the rest of diseases are due to excessive load and trauma [1–3].

Joint-related ailments are more often the concern of young and active people willing to do sports. Unfortunately, many changes in the joints have a concealed nature, without apparent discomfort. Physical activity is considered beneficial for general health, but there are reports that it may also influence the development of early osteoarthritis [4].

Osteoarthritis is a disease of the joints that involves degradation of the articular cartilage and periarticular bones. Lesions to the articular cartilage and subchondral bones are related to the development and activity of osteoclasts from the subchondral bone [4]. In the course of osteoarthritis, there are more decomposition processes than protein synthesis processes which leads to irreversible changes in the structure of the articular cartilage. As a result, degradation of proteoglycans and collagen fibres occurs [1]. This disease has a multifactorial aetiology [1, 3, 5–8].

According to recent epidemiological data, the incidence of osteoarthritis around the world varies and amounts to 2–15% of the population. In Poland, the disease affects approximately 7–8 million people; in 40% of cases, degenerative changes are located in the hip joint, and in approximately 25% in the knee joint [9].

Trace elements as cooper and zinc influence the growth, development and condition of the bone tissue [10, 11]. Changes to the mineral composition of the bone tissue can cause degenerative changes and fractures. The deficiency of certain trace elements such as zinc, selenium or copper may increase the risk of bone resorption, thus inhibiting bone growth [12].

Zinc protects the body against free radicals, stimulates metallothionein synthesis, stimulates proliferation and differentiation of osteoblasts and regulates the activity of vitamin D. Moreover, it prevents bone resorption that is stimulated by the parathyroid hormone. Zinc deficiency or its excessive loss by kidneys can lead to osteoporosis [13–15].

Copper induces low bone turnover by suppression of both osteoblastic and osteoclastic functions. It is also a co-factor of the lysyl oxidase, vital for cross-linking of collagen and elastin. The elevated concentration of copper in the serum can affect an increase in the copper stakes in the osseous tissue and the correct proprieties of the osseous tissue [16–18].

Environmental exposure to lead and cadmium is associated with the risk of occurrence of a range of chronic diseases associated with ageing, diseases of the cardiovascular system, chronic kidney failure and osteoporosis [19].

Research on the content of trace elements and components of the bone tissue concern mainly the hip joint [15, 20–25], vertebrae [26, 27] and ribs [28–31]. In contrast, research on the knee joint is quite rare [32, 33]. Therefore, it seems reasonable to undertake research on the content of elements in particular parts of the knee joint, i.e. the tibia, the femur and the meniscus.

The aim of the research was to determine the content of cadmium, nickel, copper and zinc in the tibia, the femur and the meniscus in men and women who underwent a knee replacement procedure (endoprothesoplastic surgery). The study had been selected elements of a recognised function of toxic elements (cadmium, nickel) as well as the importance of physiological (copper and zinc). An analysis of differences in accumulation of selected elements in particular parts of the hip joint was made as well due to the fact that these elements are of different tissue structure. The influence of such factors as the type of studied tissue, sex, age, place of residence (village, town), smoking, occupational exposure and changes in the content of cadmium, nickel, copper and zinc was determined.

## Materials and Methods

The study material included parts of the knee joint obtained during endoprosthesoplasty in the Dr Janusz Daab Hospital of Trauma Surgery in Piekary Śląskie. Biological samples were obtained from patients living in Silesia Province. Samples were collected from 50 patients, 36 women and 14 men. In 26 patients—the right leg and in 24 patients—the left leg were involved. The mean age of the whole study population was 67.5 years, being slightly lower in women—67.2 years than in men—68.1 years. In the study group, patients complained of pain of 10 years’ duration. A detailed description of the test group patients is shown in Table 1.

**Table 1:** Information about the study group patients

Parameters	Whole population *n* = 50	Females *n* = 36	Males *n* = 14
Age (years)			
AM ± SD	67.46 ± 7.11	67.22 ± 7.09	68.07 ± 7.20
Range	54–78	54–78	56–78
Body weight (kg)			
AM ± SD	83.54 ± 14.56	81.45 ± 14.19	88.58 ± 14.56
Range	54–115	54–115	66–108
Height (cm)			
AM ± SD	164.37 ± 9.32	160.24 ± 6.14	174.33 ± 8.11
Range	149–189	149–173	165–189
Smokers (*n*, %)			
– Non-smokers	20 (40%)	19 (38%)	1 (2%)
– Smokers	21 (42%)	10 (20%)	11 (22%)
– Smokers in the past	9 (18%)	5 (10%)	4 (8%)
Place of residence (%)			
Village	11 (22%)	7 (14%)	4 (8%)
Town	39 (78%)	29 (58%)	10 (20%)
Knee (%)			
Left	24 (48%)	18 (36%)	6 (12%)
Right	26 (52%)	18 (36%)	8 (16%)
Beginning pain (years, %)			
<5	16 (32%)	11 (22%)	5 (10%)
<10	21 (42%)	15 (30%)	6 (12%)
>10	13 (26%)	10 (20%)	3 (9%)
Earlier knee endoprosthesis (%)			
Yes	13 (26%)	10 (20%)	3 (6%)
No	37 (74%)	26 (52%)	11 (22%)
Degenerative changes in the other knee (%)			
Yes	33 (66%)	23 (46%)	10 (20%)
No	17 (34%)	13 (26%)	4 (8%)
Contact with chemicals in the workplace (factory PVC, zinc smelter) (%)	3 (6%)	1 (2%)	2 (4%)

The study was approved by the Bioethics Committee No 2/2013 of 18 June 2013. Degenerative disease of the knee joint and considerable pain were indications for this type of procedure. Surgeries were performed in subarachnoid anaesthesia, with patients in the prone position. Esmarch bandage was used for exsanguination of the limb. The frontal surface of the knee joint was exposed following standard preparation of the operation field (applying antiseptic and aseptic techniques) with straight midline incision. The joint was opened at the medial side and the hypertrophic synovium was removed. Using ZIMMER instrumentation, the femoral part of the knee joint was prepared by preparing the distal femur and performing femoral epicondyle osteotomy. Next, damaged menisci were removed, and using a Zimmer instrument, the tibial part was prepared (resection of the tibial plateau). In this way, the osseous components, cartilages and parts of menisci were used for measurements.

The material samples were described and stored in modified polyethylene containers, in a freezer, at a temperature of −22 °C.

Tissue samples with a known mass were mineralised using 4 cm^3^ of spectrally pure HNO_3_ (V) (Supra pure), Merck, in a Magnum II microwave mineraliser, Ertec. The samples were placed one by one in a Teflon vessel and added mineralisation. Mineralisation was a two-stage procedure. The first stage lasted 2 min at 20 bar maximum pressure and 255 °C maximum temperature, whereas the second stage was of 6 min duration at 45 bar maximum pressure and 285 °C maximum temperature. The post-mineralisation solution was transferred to a 25-cm^3^ flask and then diluted to the millilitre mark with redistilled water.

The content of cadmium, nickel, copper and zinc in mineralised samples was determined using inductively coupled plasma atomic emission spectrometry (ICP-AES). A Varian 710-ES spectrometer equipped with a OneNeb nebuliser was utilised. The following parameters were used: RF power, 1.0 kW; plasma flow, 15 L/min; auxiliary flow, 1.5 L/min; nebuliser pressure, 210 kPa; pump rate, 15 rpm; emission lines of Cd: *λ* = 214.439 and 228.802 nm, Ni: *λ* = 231.604 nm, Cu: *λ* = 324.754 and 327.395 nm, Zn: *λ* = 206.200 and 213.857 nm. The calibration curve method was applied. The standard solutions of 1 mg/mL (Merck Millipore, Germany) as well as deionised water (Millipore Elix 10 system) were used. The results are an average of the concentrations obtained for all analytical lines used for the element, with standard deviation not exceeding 1.5%. The accuracy of the analysis was controlled using Standard Reference Material 1400 Bone Ash (NIST).

The statistical analysis was made using the Statistica Pl. 12 software (StatSoft Crocow).

## Results

The contents of Cd, Ni, Cu and Zn in the tissues of the hip joint did not show a normal distribution, and therefore to calculate the differences, non-parametric tests were used. To assess the differences between the groups, the Mann-Whitney *U* test was used for two samples and the ANOVA Kruskal-Wallis test by ranks was used for many samples. The level of significance at *p* ≤0.05 was statistically significant.

There were statistically significant differences in the content of Cd, Cu and Zn in different parts of the knee joint (ANOVA test, *p* < 0.001). The analysis of the content of Cd in men and women showed that there is significance in differences between these groups (Mann-Whitney *U* test, *p* = 0.03). Cadmium exhibited the lowest content in tissues of the knee joint. The median corresponding to the average content of Cd in women in the tibia was 0.014 mg/kg, the femur 0.013 mg/kg and the meniscus 0.007 mg/kg. In men, the contents were slightly higher and amounted to 0.016 mg/kg in the tibia, 0.017 mg/kg in the femur and 0.008 mg/kg in the meniscus.

For Ni, there were no statistically significant differences between the studied tissues. A high amount of Ni was present in tissues of the knee joint in women compared to men. In the tibia in women, Ni was present in the amount of 0.29 mg/kg, and in men, 0.22 mg/kg. However, in the femur in women, the median for Ni was 0.36 mg/kg, and in men, 0.28 mg/kg. In the meniscus in women, it was 0.69 mg/kg, and in men, 0.42 mg/kg.

In women, the lowest Cu content was 0.36 mg/kg in the femur, then 0.39 mg/kg in the tibia and 0.65 mg/kg in the meniscus. Based on the median, the lowest content in men was in the tibia (0.31 mg/kg), followed by the femur (0.46 mg/kg) and in the meniscus (0.79 mg/kg).

The highest content of the analysed elements was observed for Zn. The median value in the femur was the highest, and in women it was 76.86 mg/kg and in men 91.68 mg/kg; in the tibia, it was 84.34 mg/kg in women and 100.35 mg/kg in men and in the meniscus it was 9.96 mg/kg in women and 10.74 mg/kg in men (Table 2).

**Table 2:** Statistical characteristics for concentration of cadmium, nickel, copper and zinc in tissues of the knee joint (mg/kg)

		Tibia *n* = 50	Femur *n* = 50	Meniscus *n* = 50
		AM ± SD	AM ± SD	AM ± SD
		Median	Median	Median
		Range	Range	Range
Cd	Men	0.019 ± 0.010	0.017 ± 0.007	0.012 ± 0.009
		0.016	0.017	0.008
		0.007–0.047	0.006–0.031	0.006–0.041
	Women	0.015 ± 0.008	0.014 ± 0.008	0.009 ± 0.006
		0.014	0.013	0.007
		0.006–0.049	0.005–0.048	0.005–0.032
	Whole population	0.016 ± 0.009	0.015 ± 0.008	0.010 ± 0.007
		0.015	0.013	0.008
		0.006–0.049	0.005–0.048	0.005–0.041
	M-W	NS	NS	NS
Ni	Men	0.39 ± 0.42	0.58 ± 0.73	0.72 ± 0.88
		0.22	0.28	0.42
		0.12–1.66	0.12–2.48	0.12–3.47
	Women	0.56 ± 0.63	0.77 ± 1.51	0.90 ± 0.85
		0.29	0.36	0.69
		0.11–3.00	0.10–8.98	0.11–3.04
	Whole population	0.52 ± 0.58	0.60 ± 0.69	0.85 ± 0.86
		0.24	0.32	0.52
		0.11–3.00	0.10–2.98	0.11–3.47
	M-W	NS	NS	NS
Cu	Men	0.45 ± 0.36	0.93 ± 1.66	0.83 ± 0.29
		0.31	0.46	0.79
		0.13–1.42	0.19–6.63	0.44–1.44
	Women	0.80 ± 1.36	0.87 ± 1.35	0.71 ± 0.32
		0.39	0.36	0.65
		0.11–6.7	0.11–5.82	0.37–1.61
	Whole population	0.70 ± 1.17	0.89 ± 1.43	0.74 ± 0.31
		0.37	0.38	0.67
		0.11–6.74	0.11–6.63	0.37–1.61
	M-W	NS	NS	NS
Zn	Men	93.53 ± 19.89	77.62 ± 41.74	21.35 ± 25.59
		100.35	91.68	10.74
		61.93–118.26	4.86–130.71	5.34–92.51
	Women	85.66 ± 20.77	82.04 ± 21.26	12.76 ± 11.94
		84.34	76.86	9.96
		17.05–128.87	49.21–130.20	5.16–78.41
	Whole population	87.86 ± 20.63	80.81 ± 28.09	15.17 ± 17.05
		88.68	79.54	10.38
		17.05–128.87	4.86–130.71	5.16–92.51
	M-W	NS	NS	NS

*AM* arithmetic mean, *SD* standard deviation, M-W Mann‐Whitney *U* test, *NS* non‐significant

**Fig. 1:**
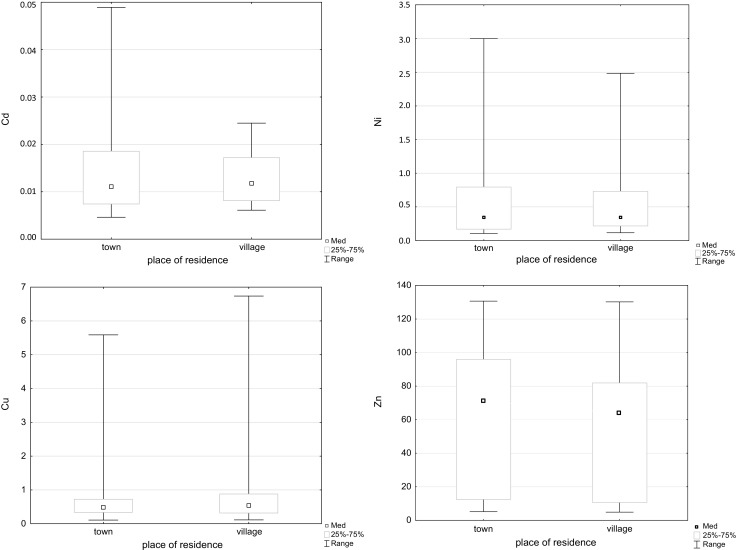
The occurrence of cadmium, nickel, copper and zinc in tissues of the knee joint, stratified by age

The studied population included people aged 54 up to 78 years and was divided into three age groups: up to 60 years old, 61–70 years old and over 71 years old. Using the ANOVA Kruskal-Wallis test by ranks for many samples, differences in the content of Cu were shown only *p* = 0.02. The largest concentration of Ni and Zn was observed in the 61–70-year age group, and in the case of Cu, the largest content occurred in the oldest age group. Differences in Cd content between the three age groups were not observed. Figure 1 shows the nature of changes in Cd, Ni, Cu and Zn in different age groups.

**Fig. 2:**
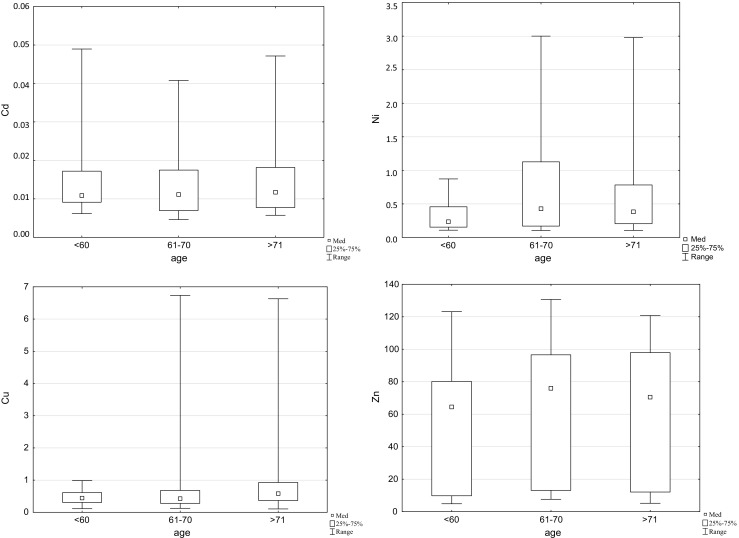
The occurrence of cadmium, nickel, copper and zinc with residents living in village and town

When comparing the content of elements in the tissues of the knee joint in patients living in rural areas and in cities, no statistically significant differences were found. Patients living in rural areas had lower contents of Cd (0.013 vs. 0.014 mg/kg), Ni (0.56 vs. 0.64 mg/kg) and Zn (56.74 vs. 62.56 mg/kg), and higher contents of Cu (1.12 vs. 0.68 mg/kg) (Fig. 2).

In the studied population, an increased content of Cd was observed in smokers (0.018 vs. 0.013 mg/kg), but these differences were not statistically significant. A statistically significant difference between smokers and non-smokers was observed for Ni (Mann-Whitney *U* test, *p* = 0.026). The content of Cu and Ni in the group of smokers was lower than in non-smokers, and for Ni, these values were 0.34 vs. 0.64 mg/kg and 0.45 and 0.75 mg/kg for Cu, respectively. The content of Zn was higher for smokers compared to non-smokers, 71.30 and 60.63 mg/kg, just as in the case of Cd (Table 3).

**Table 3:** Statistical characteristics for concentration of cadmium, nickel, copper and zinc in tissues of the knee joint (mg/kg)

	Cd	Ni	Cu	Zn	Cd	Ni	Cu	Zn
	Place of residence							
	Town (*n* = 117)				Village (*n* = 33)			
AM ± SD	0.014 ± 0.009	0.64 ± 0.69	0.68 ± 0.78	62.56 ± 39.54	0.013 ± 0.005	0.56 ± 0.56	1.12 ± 1.75	56.74 ± 40.40
Median	0.011	0.35	0.48	71.08	0.012	0.35	0.54	63.74
Range	0.005–0.049	0.10–3.00	0.11–5.59	5.16–130.71	0.006–0.024	0.12–2.48	0.11–6.74	4.86–130.20
Smoking								
	Non-smokers (*n* = 87)				Smokers (*n* = 63)			
AM ± SD	0.013 ± 0.007	0.69 ± 0.67	0.76 ± 0.98	61.67 ± 39.83	0.018 ± 0.013	0.47 ± 0.65	0.55 ± 0.30	59.89 ± 37.27
Median	0.011	0.46	0.51	70.39	0.011	0.21	0.44	71.81
Range	0.005–0.041	0.10–2.98	0.11–6.74	5.16–130.71	0.006–0.049	0.11–3.00	0.18–1.42	6.26–110.50
Contact with chemicals in the workplace								
	No (*n* = 141)				Yes (*n* = 9)			
AM ± SD	0.014 ± 0.009	0.64 ± 0.67	0.75 ± 0.94	60.63 ± 38.40	0.016 ± 0.011	0.34 ± 0.43	0.45 ± 0.25	71.30 ± 46.49
Median	0.011	0.42	0.53	70.39	0.011	0.16	0.42	71.08
Range	0.005–0.049	0.10–3.00	0.11–6.74	5.16–130.20	0.006–0.041	0.12–1.46	0.15–1.02	5.34–130.71

*AM* arithmetic mean, *SD* standard deviation

Occupational exposure may increase the content of elements in man’s tissues. Only 6% of the studied population was exposed occupationally. In those patients, there was a higher content of Zn (71.30 vs. 60.63 mg/kg) and Cd (0.016 vs. 0.014 mg/kg). However, those differences were not statistically significant. However, significance of differences was observed for Ni, where *p* = 0.07 (Mann-Whitney *U* test).

The greatest positive correlations were found between Cd–Zn, Cu–Ni and Cu–type of bones. Correlation analysis showed an antagonistic relationship between Zn–type of bones and Cd–type of bones (Table 4).

**Table 4:** Spearman’s correlation coefficients for cadmium, nickel, copper and zinc from other parameters in tissues of the knee joint

Parameters	Zn	Ni	Cu	Cd	Age	Gender	Place of residence	Knee (left, right)	Body weight	Height	BMI	Smoking
Ni	–0.11											
Cu	–0.29*	0.45*										
Cd	0.40*	–0.05	–0.01									
Type of bone	–0.69*	0.18*	0.36*	–0.39*								
Age	0.06	0.16	0.23*	–0.04								
Gender	0.06	–0.11	0.04	0.18*	0.05							
Place of residence	0.08	–0.08	–0.04	–0.19*	–0.07	–0.22*						
Knee (left, right)	0.06	–0.01	0.14	0.03	0.06	–0.06*	0.19*					
Body weight	–0.03	0.00	–0.05	0.02	–0.40*	0.20*	–0.19*	0.01				
Height	0.04	0.01	0.05	0.13	–0.01	0.68*	–0.36*	–0.16	0.52*			
BMI	–0.03	0.00	–0.05	0.02	–0.40*	0.20*	–0.19*	0.01	1.00	0.52*		
Smoking	0.00	0.21*	0.04	–0.10	0.15	–0.31*	–0.13	0.04	0.02	–0.11	0.02	
Beginning pain	–0.10	0.07	0.05	0.01	0.14	–0.03	–0.04	–0.17*	–0.17	–0.10	–0.17	0.16

(*p < 0.05, statistically significant

## Discussion

The use of human tissues, especially those that are acquired in the course of surgeries and are considered medical waste, becomes more frequent. The assessment of the content of metals in bone tissue is used by many researchers to determine the level of exposure. The accumulation of metals is illustrated in the mineral composition of bones, and in the case of some elements such as cadmium and lead, it also indicates the level of exposure in the past. Due to their structure, bones are characterised by a very slow rotation of elements which biological half-lives are estimated to be approximately several to several tens of years [15, 34–37]. The content of elements in bones depends on several factors, including age, sex, place of residence, health status, smoking or diet.

Among the elements tested, Cu and Ni showed a high content in the connective tissue (meniscus) compared to the bone tissue (tibial and femur). Cu plays an important role in the process of production of collagen; therefore, its content in both the bone and connective tissues is high [38–40]. Ni is mainly found in soft tissues; however, its presence in the osseous tissue has also been confirmed. This element influences the skeleton metabolism. In animals, Ni causes growth disorders and contributes to marrow hyperplasia [41].

Comparing the content of elements in tissues of the knee joint, for example, with the content in ribs, there is a higher content of Cd, Ni, Zn and a lower content of Cu [42]. According to Zaichick et al. [29, 30, 43], ribs show higher contents of Cd, Ni and Cu, and the content of Zn in the tibia and the femur was at a similar level.

In case of nickel, most of this element was in the meniscus. However, differences in the content of nickel in different parts of the knee joint are not statistically significant. This means that most of Ni is accumulated in the connective tissue rather than in the bone tissue. In women, in the tibia there is 38% less Cu and in the femur 15% less Cu compared to the meniscus. In men, the content of Cu in the tibia was 46% lower and in the femur 20% lower compared to the meniscus.

It was observed that the hip joint showed a 100% higher content of Cd compared to the knee joint. Łanocha et al. [20] inform that the content of Cd in the cortical bone and the articular cartilage of the hip joint in women was at the level of 0.026, and in men, 0.027 mg/kg. This result was similar to the research by Bush et al. [39] (0.029 mg/kg). Lanocha et al. [42] observed that Cd concentration in the cortical bone and the articular cartilage of the hip joint did not exceed 0.031 mg/kg dw. Moreover, there were no significant differences in the concentrations of Cd between smokers and non-smokers [42].

Based on the conducted studies, it can be observed that the bone tissue had approximately 60% more Cd compared to the connective tissue—the meniscus. The content of Cd in the body is generally higher in women than in men, and this is due to increased gastrointestinal absorption at lower concentrations of iron. However, the conducted studies did not show statistically significant differences in the content of Cd between men and women: 0.016 and 0.013 mg/kg ww.

In case of Cu, its content in particular parts of the hip joint was statistically significant (ANOVA test, *p* < 0.001). Based on the median value, the content of Cu was highest in the meniscus, which is quite distinctive and specific. The content of Cu was at a very similar level, which in our study was 0.78 mg/kg and in the research on the hip joint by Lanocha et al. [19, 42] averaged to a value of 0.79 mg/kg. Zioła-Frankowska et al. [44] observed slightly higher Cu concentration (0.90 mg/kg) than in our research (0.78 mg/kg). Garcia et al. [45] found that Cu level in bones did not exceed 1.53 mg/kg.

The highest content of Zn in both women and men was found in the tibia and the lowest in the meniscus; in women, the content of Zn was higher by 85%, and in men, by 77%. In case of the content of Zn in selected tissues of the knee joint, there were statistically significant differences (ANOVA test, *p* < 0.001).

The average content of Zn in the tibia marked by Lanocha-Arendarczyk et al. [31] was at a similar level at 98.90 mg/kg when compared to our results (87.86 mg/kg). Similarly, there were no statistically significant differences in the content of Zn in men and women, whereas in men they were slightly higher [31].

The content of Cd in three age groups was at the same level, which may suggest that the reserve of Cd in human body is subject to constant exchange and no long-term accumulation is present.

The content of Cd in bones from the industrialised region of Tarragona (Spain) was determined by Garcia et al. [45] to be 0.025 mg/kg, and there were no statistically significant differences between men and women as well as smokers and non-smokers.

Lanocha-Arendarczyk et al. [31], in patients from NW Poland after knee surgery, reported a higher Cd concentration in men than in women, which is similar to our research. Women’s tendency to a greater accumulation of Cd in comparison with men (0.06 vs. 0.04 mg/kg), which is often quoted in literature [28, 30, 46], was not confirmed. The content of Cd indicated by Lanocha-Arendarczyk et al. [31] was higher than our results (0.05 vs. 0.016 mg/kg). There was a higher content of Cd in the group of smokers compared with non-smokers (0.06 vs. 0.03 mg/kg), as also confirmed by the research by Lanocha-Arendarczyk et al. [31].

According to Lanocha-Arendarczyk et al. [46], the content of Ni in the hip joint was 0.177 mg/kg, and this value was significantly lower compared to the content in the knee joint (0.76 mg/kg). The content of Ni in occupationally exposed and unexposed population in the research by Lanocha-Arendarczyk et al. [46] was variable depending on the type of the examined tissue. In the articular cartilage, as in our research, the significance of differences was at a similar level; in the cortical bone, the content of Ni was higher in the unexposed population, and in the cancellous bone, in the occupationally exposed population. In the hip joint, the content of Ni identified by Zioła-Frankowska et al. [44] was similar to 0.70 mg/kg.

The content of Ni in bones from people living in industrialised areas of Spain was 1.20 mg/kg [45]. Similarly to the results obtained (0.80 and 0.64 mg/kg), the content of Ni was higher in women (1.474 mg/kg) than in men (1.20 mg/kg) [41].

The content of Zn in the tissues of the hip joint in inhabitants of the north-western region of Poland [19, 42] was as follows: the cortical bone with the articular cartilage and cancellous bone—88.29 and 83.10 mg/kg dw. The content in the knee joint was at a similar level: the tibia 87.86 and the femur 80.81 mg/kg. Slightly lower values of Zn were reported by Zioła-Frankowska et al. [44], and this concerned the hip joint of people from the Greater Poland Province; in the cortical bone it was 72.09 mg Zn/kg, and in the cancellous bone, 68.7 mg Zn/kg. In the research by Brodziak et al. [15], the content of Zn in the cortical bone was at a similar level as in the knee joint.

According to research on the territory of Spain published by Garcia et al. [45], the content of Zn in bones was 39.40 mg/kg.

Cd and Zn are among the elements with proven antagonistic relations because Cd ions inhibit intestinal absorption of Zn among others [15, 47]. The conducted correlation analysis between the two elements showed significant synergistic correlations between Cd and Zn (*r* = 0.40, Spearman *p* < 0.05). Nickel may have both synergistic and antagonistic correlations with most elements, whereas Cu correlates antagonistically with Zn [47]. In the obtained research results, nickel correlated with Cu (*r* = 0.45). For Cu, antagonistic relations between Cu and Zn were confirmed (−0.29). Cd–Zn correlations were not confirmed by Lanocha et al. [31] and Kuo et al. [48]. In the research by Kuo et al. [48], the same correlation as in our research—Ni–Cu (*r* = 0.57)—was observed. The correlation between nickel and Cu was confirmed in the research by Zioła-Frankowska et al. [44] both in the cortical bone (*r* = 0.57) and the cancellous bone (0.44).

## Conclusions

There were statistically significant differences in the content of cadmium, copper and zinc between the examined tissues, i.e. the tibia, the femur and the meniscus. Among the elements tested, copper and nickel showed a high content in the connective tissue (the meniscus) compared to the bone tissue (the tibia and the femur).

There were no statistically significant differences in the content of cadmium, nickel, copper and zinc in women and men in the examined parts of the hip joint.

One of the most common correlations described in literature that was confirmed is the synergistic correlation between nickel and copper.

The content of cadmium in tissues of the knee joint was significantly lower as compared with the hip joint, and the content of zinc was at a similar level.

The content of cadmium in three age groups was at the same level, which may suggest that the reserve of cadmium in the human body is subject to constant exchange and no long-term accumulation is present.

The population of occupationally exposed people showed higher contents of zinc and cadmium.
